# Conservation of the Eastern Taiwan Strait Chinese White Dolphin (*Sousa chinensis*): Fishers' Perspectives and Management Implications

**DOI:** 10.1371/journal.pone.0161321

**Published:** 2016-08-15

**Authors:** Ta-Kang Liu, Yu-Cheng Wang, Laurence Zsu-Hsin Chuang, Chih-How Chen

**Affiliations:** 1 Institute of Ocean Technology and Marine Affairs, National Cheng Kung University, Tainan City, Taiwan; 2 Department of Law, National Cheng Kung University, Tainan City, Taiwan; Institute of Deep-sea Science and Engineering, Chinese Academy of Sciences, CHINA

## Abstract

The abundance of the eastern Taiwan Strait (ETS) population of the Chinese white dolphin (*Sousa chinensis*) has been estimated to be less than 100 individuals. It is categorized as critically endangered in the IUCN Red List of Threatened Species. Thus, immediate measures of conservation should be taken to protect it from extinction. Currently, the Taiwanese government plans to designate its habitat as a Major Wildlife Habitat (MWH), a type of marine protected area (MPA) for conservation of wildlife species. Although the designation allows continuing the current exploitation, however, it may cause conflicts among multiple stakeholders with competing interests. The study is to explore the attitude and opinions among the stakeholders in order to better manage the MPA. This study employs a semi-structured interview and a questionnaire survey of local fishers. Results from interviews indicated that the subsistence of fishers remains a major problem. It was found that stakeholders have different perceptions of the fishers’ attitude towards conservation and also thought that the fishery-related law enforcement could be difficult. Quantitative survey showed that fishers are generally positive towards the conservation of the Chinese white dolphin but are less willing to participate in the planning process. Most fishers considered temporary fishing closure as feasible for conservation. The results of this study provide recommendations for future efforts towards the goal of better conservation for this endangered species.

## Introduction

The Chinese white dolphin, also named as Indo-Pacific humpbacked dolphin (*Sousa chinensis)*, can typically be observed in coastal waters from Southeast Asia to northern Australia [1–4]. Globally, the population of the Indo-Pacific humpbacked dolphin is likely in the range of 10,000–100,000. Although this species is classified as Near Threatened (NT) in the Red List of Threatened Species published by the International Union for Conservation of Nature (IUCN) [5], the population living in western Taiwan's waters was claimed to be significantly different in pigmentation from those populations in Xiamen and Hong Kong waters, but no significant evidence from genetic analysis thus far [6–10]. The number of ETS Chinese white dolphins is estimated below 100 individuals and the population is declining [11]. Based on a photo-identification field survey during 2007–2010, 71 individuals were identified [10, 12]. As a result, they are classified as Critically Endangered (CR) in the IUCN’s Red List of Threatened Species [13].

The ETS Chinese white dolphins have been claimed to be facing five main threats: the degradation or disappearance of their habitat, underwater noise, the decrease of land-based discharged freshwater, pollution, and fishing activities [14–15]. In recent years, with the increasing problems of overfishing and illegal fishing, the awareness of marine conservation has gained in importance. Numerous countries have designated marine protected areas (MPAs) in response to the global problem of overfishing and species extinction. MPAs intended primarily to protect the Chinese white dolphin have also been established, such as in Xiamen and Zhuhai, China, and Sha Chau and Lung Kwu Chau Marine Park in Hong Kong for the protection of Chinese white dolphins [6, 15–19].

**Fig 1:**
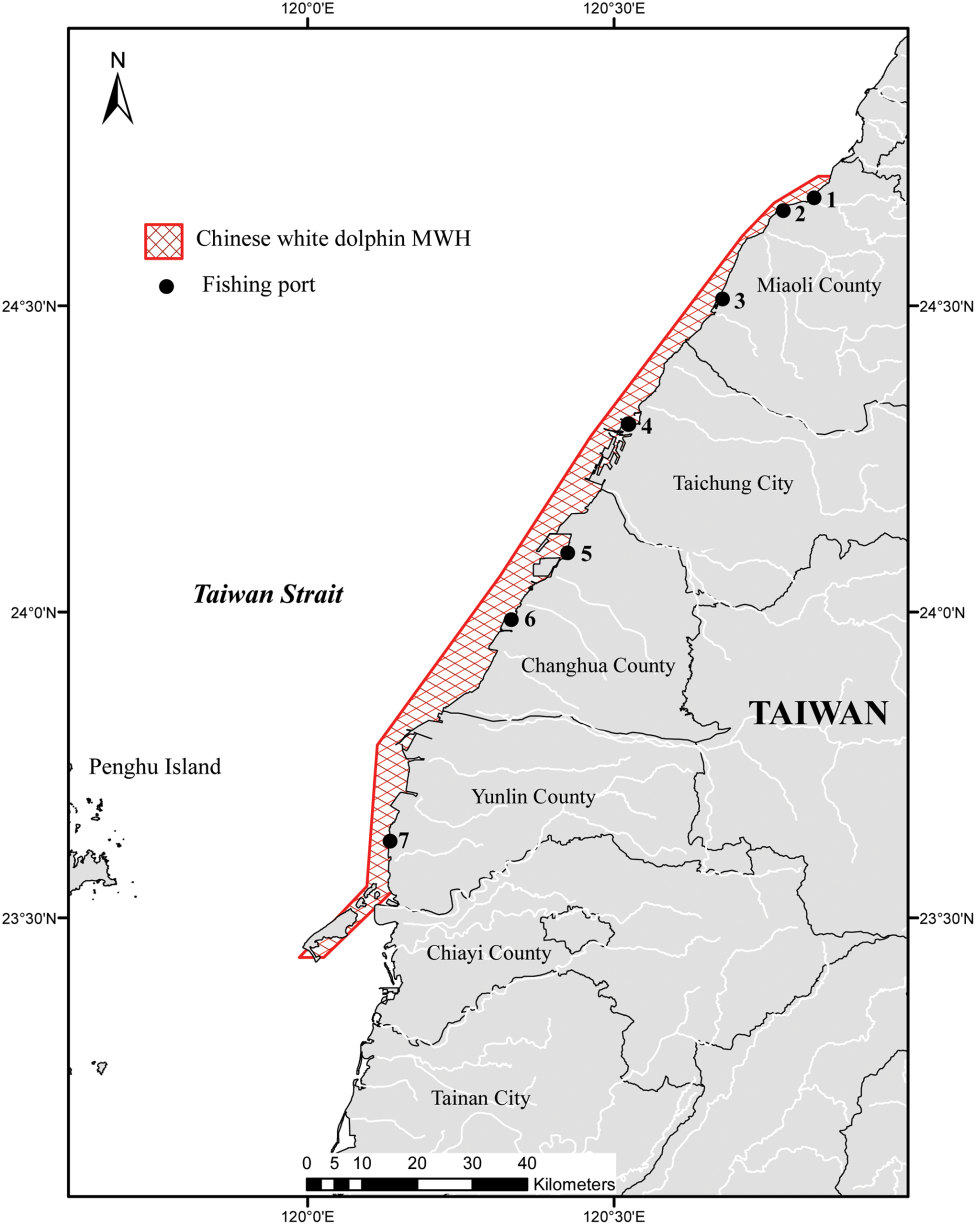
The proposed Chinese white dolphin Major Wildlife Habitat (MWH) in Taiwan. Fishing ports: 1. Long-feng; 2. Wai-pu; 3. Tong-shiao; 4. Wu-chi; 5. Lun-wei-wan; 6. Wang-gong; 7. Bo-zi-liao.

Taiwan’s Council of Agriculture has announced the Protected Species in 2008. The ETS Chinese white dolphin is listed as a Category-I Endangered Wildlife (i.e., the most critical species). In order to ensure the survival of the ETS Chinese white dolphin, its activity and habitat range must be designated as an MPA to conserve its living environment. In Taiwan’s Wildlife Conservation Act, MPAs have two designations based on the degree of the protection, i.e., major wildlife habitat (MWH) and wildlife protected area (WPA). In 2011, conflicts between the proposed Chinese white dolphin MWH and the Kuo Kuang Offshore Industrial Zone led to the cancellation of development in the industrial zone [20]. Because Taiwan lacks practical experience in the conservation of marine mammals, further regulatory and enforcement difficulties along with rights disputes with stakeholders may be encountered when establishing an MPA for the Chinese white dolphin in the future. In particular, the proposed Chinese white dolphin MWH (Fig 1) overlaps with coastal fisheries such as gillnets that could be restricted due to potential harm to the Chinese white dolphin. Our previous study indicates that the feelings and views of fishers regarding the MPA are certain to be key factors in the success of its designation [20]; fishers are typically profoundly affected by the conflicts of interest inherent in the current depletion of marine living resources and species conservation. In this study, qualitative interviews were performed first to explore the opinions of the fishers and other potential stakeholders towards the conservation of the ETS Chinese white dolphins. It aimed to seek the extent of their support for and their insights regarding marine conservation and to explore whether the designation of the MPA would result in conflict and misunderstanding. Secondly, questionnaires were designed based on the findings from the interview and employed for deeper investigation in this study. The results can serve as future recommendations for the planning process and subsequent management of a successful MPA.

## Research Method

### Study area

The study area covered the cities and counties within the boundaries of the Chinese white dolphin MWH currently being proposed by the Forestry Bureau, Council of Agriculture. From north to south, these cities and counties comprise Miaoli County, Taichung City, Changhua City, and Yunlin County (Fig 1). Qualitative interviews and a questionnaire survey were conducted in this study. With the exception of the interviewees from the central government agencies, all of the interviewees’ work locations were within the coastal cities along the habitat of the Chinese white dolphin. Fishery statistical data published by the Fisheries Agency were used as a screening tool for the questionnaire survey [21]. Fishing ports with relatively high fisheries output in the cities and counties within which the Chinese white dolphin appears were the primary survey locations.

### Ethics statements

This study was conducted back in 2011 when qualitative research and questionnaire survey does not require approval of institutional review board (IRB) in Taiwan. In this study, the qualitative interview and questionnaire survey were conducted by the authors. Before the interview or questionnaire, we introduced ourselves and explains the scope of this study. We explained why the respondent has been selected and what will happen to the data collected. The participants had understood the purpose and details of the study and had voluntarily agreed to be interviewed. When recording started, the researcher asked again if the respondent is willing to be interviewed to document the verbal consent.

### Qualitative interviews

Semi-structured interviews were conducted to examine fishery-related stakeholders’ perceptions of the proposed Chinese white dolphin MPA. A set series of questions related to the MPA was presented to the interviewees with topics intended to be explored, such as fishers’ perceptions towards the MPA, the effectiveness of the MPA, current EST Chinese white dolphin-related fishery issues, the balance between coastal development and conservation, and the role and future efforts of the competent authorities. The interviews were conducted in a relaxed conversational manner and the flexibility of the questions was also maintained to allow the interviewees to fully elaborate their interpretations of the controversial issues [22]. Due to the nature of the multiple uses of the marine environment, numerous stakeholders may be involved during the process of MPA planning [23]. Purposeful sampling was typically employed to choose the interviewees in semi-structured interview. Theoretically, when seeking to elicit an inventory of the concepts of a population, it is possible to identify nearly all of them from a diverse sample of ~20 individuals [24]. In this study, fishers and the key officers from the fishers’ associations were the main stakeholders explored. Other distinctive stakeholders, included scholars and experts at marine-related academic institutions, the members from competent authorities responsible for designating critical habitats, and non-governmental environmental groups.

### Questionnaire survey

The questionnaires were conducted by the authors and distributed at seven fishing ports adjacent to the ETS Chinese white dolphin MWH. Names and locations of the fishing ports were exhibited in Fig 1. Fishers were asked to complete the designed questionnaire. The targeted fishers were those who can recognize the Chinese white dolphins. In order to obtain a sufficient number of appropriate subjects, the sampling methods for the questionnaire were snowball sampling and purposive sampling. When the questionnaire was administered, photographs of the Chinese white dolphin were provided for the tested fishers to view to assure that the respondents actually know the species in questionnaire. Statistical Product and Service Solutions (SPSS) ver. 17.0 was used for descriptive statistics analysis. Chi-square testing was used in to explore whether fishers’ opinions depended on their fishery related characters, i.e., fishing technique. The questionnaire involves several categories of questions, including demographic data, experiences of interacting with the Chinese white dolphin, influence of the Chinese white dolphin on the fishers, and perceptions towards the conservation of the Chinese white dolphin and fishery resources. Detailed questionnaires can be seen in the Supporting Information file (S1 Text).

## Results

### Qualitative interview results

A total of 21 individuals from various stakeholder groups were interviewed, consisting of 6 people from various fishers’ associations, 5 fishers, 3 people from fishery-related governmental agencies, 2 scholars (one marine mammal biologist and one fishery economist), and 5 people from environmental groups. The results of our analysis indicated that only the respondents from the central authorities and some people from environmental groups and academia had more grasp of this issue. The officers and fishers from the local fisher’s associations and the officials in local government agencies seemed unfamiliar on the issue of the designation of the Chinese white dolphin MWH. Because the local governmental fishery authority and fisher’s associations have yet to be consulted regarding the designation of the MPA, they perceived that they were not participating in this process and that their views were being neglected. Thus, it is recommended that the competent authority in central government should conduct more public hearings and briefings to facilitate dialogue with the local levels and achieve effective communication and coordination. The environmental groups and scholars held that the MPA should be designated as quickly as possible since the population of Chinese white dolphins is already extremely low. Most of the interviewees from fishers’ associations indicated that the designation of the Chinese white dolphin MWH seemed necessary from the stance of conservation, but their support was conditional, i.e., fishing activities and the livelihoods of the fishers must not be compromised. If these conditions could not be maintained, they tend to be doubtful and think MPA would lead to protests among fishers. Some of these respondents were opposed to the MPA. They mentioned that once the MPA is established, the number of rules and restrictions is bound to increase and thus fishing activities are sure to be strictly regulated.

One scholar indicates that scientific evidence exists for the competition for fishery resources between the Chinese white dolphin and fishers. The primary target species of gill nets and pole and line fishing has a high degree of overlap with the prey of the Chinese white dolphin [25–26]. This is generally considered to be an important reason behind the opposition of the Chinese white dolphin MPA among fishers. The majority of the interviewees believed that obtaining a balance between the conservation of the Chinese white dolphin and the livelihoods of the fishers should involve more communication with fishers and fishers’ associations during the MPA planning process. In addition, the competent authorities must spend more time and efforts on coordination and advocacy, and should explore in detail the compensatory measures for fisheries. Most of the interviewees held that fishers could accept and cooperate with the competent authorities on the MPA if enough time were spent on communicating with them and continued advocacy. A number of the interviewed fishers stated that fishing is not the primary factor behind the decline in fishery resources. Rather, they indicated that the primary factor is the substantial influence of coastal industrial development and pollution on fishery resources. The survival of Chinese white dolphin is threatened since coastal pollution reduces their food sources.

Since Taiwan has not designated any MWHs for marine mammals, the conservation of cetaceans remains in an unfamiliar issue for the competent authority. If an MWH is designated for the Chinese white dolphin in the future, it will be the first marine mammal to be protected under the Wildlife Conservation Act. Because the initial legislative purpose of the Wildlife Conservation Act was primarily the protection of land-based wildlife in the forests, the Forestry Bureau under Council of Agriculture is conveniently assigned as the competent authority for this statute. A number of the interviewees from the fishers’ associations expressed concerns regarding the position of the Forestry Bureau as the competent authority for the conservation of the Chinese white dolphin. They stated that the Forestry Bureau’s professionalism with regard to the conservation of marine mammals is likely weaker than that of the Fisheries Agency. The Fisheries Agency possesses more knowledge and experiences in fishery related issues. Thus, management would be more appropriate in the hands of the higher-level Council of Agriculture, since both the Forestry Bureau and Fisheries Agency are under this ministry. Some of the interviewees suggested that upgrading the level of the competent authority to ministry-level Council of Agriculture may benefit the conservation of the Chinese white dolphin. In addition, this measure help tighten inter-ministerial cooperation. In particular, the future implementation of conservation measures for the Chinese white dolphin will depend on the enforcement of the relevant laws by the Coast Guard Administration. The sound cooperation and coordination between central governmental agencies can result in enhanced management capabilities, improving the effectiveness for the proposed conservation measures for Chinese white dolphin.

### Questionnaire survey results

The questionnaire survey was administered from March 2012 to April 2012. The education level of the fishers was generally relatively low, so the researchers assisted them in completing the questionnaire. The fishers were typically busy that once in the fishing port areas, they were engaging in their fishery related works and may refuse to participate in our survey. Therefore, a total of 200 fishers were approached and only 103 questionnaires were distributed. After removing those who were unable to complete the whole questionnaire, the valid number of respondents to questionnaire was 97. Table 1 showed the demographic data that the ratio of men was relatively high. The majority of the female fishers also refused to respond because they seldom went out to sea. They helped maintain the fishing gears or sell catches instead. Approximately half of the respondents had at least 20 years of extensive experience. This phenomenon is also consistent with the severe aging trend of fishers currently observed in the fishing ports, with 76.3% of the responding fishers being at least 50 years of age.

**Table 1:** Demographic data of the respondents in the questionnaire survey (n = 97).

Items		Number
Gender	Male	96
	Female	1
Experience	< 5 years	4
	5–10 years	11
	10–15 years	17
	15–20 years	13
	20–25 years	15
	> 25 years	37
Age	30–39 yo	2
	40–49 yo	21
	50–59 yo	45
	> 60 yo	29
Vessel	Sampan	25
	PVC pipe raft	56
	Tonnage < = 5	9
	Tonnage 5–9	1
	Tonnage > = 10	6
Fishing technique	Gillnet	62
	Trawling	6
	Pole and line	27
	Other	2

#### The fishers’ interactions with the Chinese white dolphin

Table 2 shows more than half of the respondents held that the number of Chinese white dolphins had not changed much. Most of them who thought so stated that the chance of the Chinese white dolphin being sighted is rather low, so they lacked deep feelings of it. The others indicated that in comparison with their past experience, the number of times they encountered the Chinese white dolphins was clearly falling. Table 2 indicates that the respondents witnessed the white dolphin relatively frequently from April to June and from July to September.

**Table 2:** Fishers' opinion about the Chinese white dolphin population and their sighting frequency (n = 97).

Item		Frequency	Percentage (%)
Feeling about the fluctuation of the Chinese white dolphin population	Increase a lot	3	3.3
	Increase slightly	1	1.1
	No Change	40	44.4
	Decrease slightly	13	14.4
	Decrease a lot	33	36.7
The frequency that that Chinese white dolphins were sighted	Jan ~Mar	27	10.4
	Apr ~ Jun	136	52.0
	Jul ~Sep	68	26.1
	Oct ~ Dec	13	4.9
	Cannot recall which month	17	6.5
	Total	261	100.0

#### The impact of the Chinese white dolphin on the fishers

Table 3 shows that during offshore operations, 76.7% of the fishers held that their landings dropped when Chinese white dolphins are actively present nearby. Officials from the fishers’ associations and the government units also noticed that fishers and the Chinese white dolphins compete for the same fishery resources. Table 3 shows 81.4% of the fishers held that no correlation existed between the predatory behavior of Chinese white dolphin and the gradual decline of fishery resources in general, and 87.6% of the respondents had never heard of bycatch of Chinese white dolphins. Since the number of the Chinese white dolphin is so low, it is likely that fishers would not place blame for the overall decline in fishery on the predation and Chinese white dolphins, and most of them never heard of a bycatch.

**Table 3:** Fishers' opinion about the impact of the Chinese white dolphin on the fishery (n = 97).

Items		Frequency	Percentage (%)
Effect on landings when Chinese white dolphin is nearby	No change	21	23.3
	Decrease slightly	17	18.9
	Decrease a lot	52	57.8
Relation between the gradual decline of fishery resources in general and foraging of Chinese white dolphin	Significantly related	1	1.0
	Related	11	11.3
	No comment	6	6.2
	Not related	37	38.1
	Significantly not related	42	43.3
Ever heard of bycatch of the Chinese white dolphin	Sometimes	3	3.1
	Rare	9	9.3
	No	85	87.6

#### Opinions on the conservation of Chinese white dolphins

Table 4 shows that 76.3% of the respondents approved the conservation of Chinese white dolphin. Those who approved were queried further regarding the reasons for their approval. These reasons can be summarized with three points: (1) They are already legally designated protected species; (2) The number of Chinese white dolphins is low and they must be conserved for our future generations; (3) They are part of the ocean and their existence should be preserved. Some fishers holding opposing views indicated that because of the promotion of dolphin conservation, the overall number of dolphins is already too high, and that further conservation measures should not be pursued. Table 4 shows that 48.4% of the respondents indicated that the views of fishers are critical during the planning of Chinese white dolphin MPA. The majority of the rest stated that the planning of MPA has no direct relationship with fishers, so their participation is not necessary. This is close to the willingness of fishers to assist in the conservation efforts that 38.2% of the respondents stated yes. Table 4 also shows 58.8% of the fishers were willing to participate in public hearings, whereas 41.2% had no opinion or were unwilling to participate. Upon further query, the majority indicated that they lacked confidence in government agencies and believed that the positions of fishers would be ignored. In addition, the working hours of fishers may not allow them to attend.

**Table 4:** Fishers' attitude towards the conservation of Chinese white dolphin (n = 97).

Items		Frequency	Percentage (%)
Support for the conservation of Chinese white dolphin	Strongly agree	30	30.9
	Agree	44	45.4
	No opinion	19	19.6
	Oppose	3	3.1
	Strongly oppose	1	1.0
Whether fishers should participate in the planning of Chinese white dolphin MPA	Strongly agree	21	21.6
	Agree	26	26.8
	No opinion	23	23.7
	Disagree	21	21.6
	Strongly disagree	6	6.2
Willingness to work for the conservation of Chinese white dolphin	Strongly agree	5	5.2
	Agree	32	33.0
	No opinion	26	26.8
	Disagree	30	30.9
	Strongly disagree	4	4.1
Participate in the hearing or workshop related to the conservation of Chinese white dolphin	Agree	57	58.8
	No opinion	35	36.1
	Disagree	5	5.2

Table 5 shows the cumulative percentages of the respondents who were willing to cooperate on the various conservation measures. The results indicate that 62.9% of the fishers would approve seasonal fishery closure. Nearly half of the fishers agreed with the other measures, such as no-take reserve for ecologically significant core zones and restriction on mesh size of fishing nets. Only 7.2% of the fishers were unwilling to accept any of the conservation measures, indicating that the majority of the respondents could accept measures to facilitate the restoration of fishery resources.

**Table 5:** Fishers' opinion related to feasible fishery management and the possible fishery restrictions they approve for the conservation of Chinese white dolphin (n = 97).

Fishery restrictions	Frequency	Percentage (%)
Set up no-take zone for ecologically significant area	49	50.5%
Seasonal closure	61	62.9%
Total allowable catch	13	13.4%
Fishing gear restriction	48	49.5%
Other measures	10	10.3%
Disapprove the above	7	7.2%
Total	188	193.8%

#### The effects of fishing techniques on the fishers’ opinions

Table 6 shows the use of chi-square testing to explore whether the fishers’ opinions depended on their fishing techniques, e.g., pole and lines and gill nets. The operating ranges of these fishing techniques tend to be along coastal areas, with considerable overlap with the habitat of the Chinese white dolphin. Table 7 showed the two-way table and chi-square value for the hypothetical questions in Table 6. It is noted that the willingness to assist in the conservation of the Chinese white dolphin varied significantly depending on fishing technique (p = 0.001, < 0.05), as seen in Test 3 in Table 6. Additionally, acceptance of restrictions on gill nets (Test 4) also differed significantly (p = 0.000, < 0.05). The ratio of fishers who used gill nets and were willing to assist was relatively low at 43.5%, whereas the ratio of fishers who used pole and lines and were willing to assist was relatively high at 66.7%. That acceptability of restrictions on gill nets varied significantly (p = 0.00, < 0.05) is predictable. Restrictions on the use of gill nets would have relatively little effect on other fishing techniques. Thus, 85.2% of the fishers who used pole and lines accepted such restrictions. In contrast, 67.7% of the fishers who used gill nets were opposed to these restrictions. Some of the fishers who used gill nets indicated that they could accept restrictions on gill nets in ecologically important areas designated by scientific investigation, if such restriction may help the recovery of declining fishery resources.

**Table 6:** Statistical hypothesis testing for fishers using different fishing techniques and their opinion regarding the conservation of Chinese white dolphin (n = 62).

Test	Null hypothesis	Significance (p < 0.05)
1	H_0_: Fisher using different fishing techniques may have the same opinion regarding to the planning of Chinese white dolphin MPA.	Not Reject H_0_ (p = 0.811)
2	H_0_: Fisher using different fishing techniques may have the same opinion in their willingness to participate in the public hearing related to the planning of Chinese white dolphin MPA.	Not Reject H_0_ (p = 0.488)
3	H_0_: Fisher using different fishing techniques may have the same opinion in their willingness to assist in the conservation of the Chinese white dolphin.	Reject H_0_ (p = 0.001)
4	H_0_: Fisher using different fishing techniques may have the same opinion for restricting the use of the gillnet for the conservation of Chinese white dolphin.	Reject H_0_ (p = 0.000)
5	H_0_: Fisher using different fishing techniques may have the same opinion regarding other fishers' compliance to the conservation measure.	Not Reject H_0_ (p = 0.363)

**Table 7:** Differences in views among the fishers who employed different fishing techniques (n = 62).

Opinion of fisher using different fishing techniques		Gillnet	Pole and line	Chi-square
Fishers’ involvement of discussion before the planning of Chinese white dolphin MPA	Yes	30	15	0.42
	No comment	15	6	
	No	17	6	
Willingness to participate in the public hearing related to Chinese white dolphin MPA	Yes	35	18	1.43
	No comment	24	7	
	No	3	2	
Willingness to assist in the conservation of the Chinese white dolphin	Yes	18	18	14.14
	No comment	17	7	
	No	27	2	
Opinion for restricting the use of the gillnet	Approve	15	23	35.53
	No comment	5	4	
	Oppose	42	0	
Other fishers will comply to the conservation measures of the government	Yes	27	10	2.03
	No comment	14	10	
	No	21	7	

## Discussion

Proper public participation that broadly incorporate the opinions of all stakeholders and effective environmental education for ocean users, such as fishers or the general public, during the planning of an MPA would facilitate the future management efforts. Great Barrier Reef Marine Park in Australia was noted the as one successful example [27]. The process of establishing a protected area in North Central California in the United States was also a successful case of public participation [28]. Because Taiwan currently lacks experience in designating MPAs for cetaceans, dialogue between government agencies and stakeholders and public participation are crucial to the planning of the Chinese white dolphin MWH. The qualitative interviews indicate that the interviewees from the fishers’ associations and the fishers themselves, and officers from local governmental fisheries affairs were not well informed of this issue. Officers at the Fisheries Agency held that a final decision on the proposed MPA must be made before advocacy begins. On the contrary, the majority of the stakeholders held that early engagement of public hearings or procedures for communication and coordination during the planning process would help marine resources users to understand the intentions of the designated MPA. It was noted that there is a communication gap between central and local authorities. The addition of channels for communication before designation would help local governmental agencies to provide recommendations, reducing the potential for controversy within the designated ranges and facilitating conservation works.

In this study, the Chinese white dolphins were more frequently sighted from April to June and from July to September. It was noted that in the waters of Hong Kong, the number of groups of Chinese white dolphins does not vary significantly with the seasons [6]. The frequency with which Chinese white dolphins appear in the waters of Algoa Bay in South Africa is also not significantly correlated with the seasons [29]. Studies have indicated that because of the influence of the winter northeast monsoon in Taiwan, the poor climate may have limited the sea observations of the Chinese white dolphin [30]. In summary, it is likely that the respondents in this study conduct fishing operations primarily during the summer when the weather is relatively stable, so the chance of sighting increased during that time period.

Analysis of the interview content from the fishers’ associations and the fishers indicates that the livelihoods of the fishers were naturally their primary consideration. The factor behind doubts and opposition towards the designation of the MPA was mainly the concerns over whether fishing activities would be restricted. Although this MWH is a passive MPA at the planning stage, however, most fishers worry about that the managing authority can implement striker fishery restriction that is considered necessary to conserve the Chinese white dolphin according to the Wildlife Conservation Act once the MWH is established. The opposition of fishers seemed to originate from their concerns with such uncertain conservation measures in the future. Therefore, communication with fishers should be increased to clarify the conservation measures, which should reduce the din of opposition. Fishery resources are currently in decline, so the marine environment needs to be protected for the recovery of living resources. Obtaining the approval of fishers would be a major boost to the governance of a sustainable marine environment. If some fishing techniques are to be restricted in the future to protect the Chinese white dolphin, scientific investigation is necessary to study the interrelation between the Chinese white dolphin and gill nets first. The data obtained from the interviewed fishers indicate that the views of the respondents regarding the impact of gill nets on the white dolphin varied. Currently, the literature indicates that more than 30% of Taiwan’s Chinese white dolphins have wounds and scars, which may be caused by fishing gear or collisions with ships [11, 31]. However, direct evidence indicating that Chinese white dolphins get caught accidentally in gill nets has yet to be investigated.

### Implications from the survey

The responding fishers in this study had positive attitudes towards the conservation of the Chinese white dolphin, with 76.3% approving of the proposed conservation efforts. Most fishers (76.7%) indicated that their landings dropped when the Chinese white dolphins were actively present nearby. This is consistent with the results from Taiwan’s northeastern fisheries that the appearance of cetaceans significantly reduces the operating incomes of fishing vessels using longlines and trolling lines in Nanfangao waters [32]. However, 81.4% of the respondents did not believe that the decline in fishery resources is connected to the predation of Chinese white dolphins. They stated that the predation of the Chinese white dolphin does not have a substantial influence, and that for cetaceans to prey on fish should be regarded as a normal ecological phenomenon. The majority of the respondents stated that the decline in fishery resources was primarily due to illegal fishing, ecological damage caused by bottom trawling, and industrial pollution. These were also potential problems affecting the food sources of the white dolphin. Overall, the fishers had friendly perceptions towards the Chinese white dolphin.

The results of questionnaire showed that the difference in fishers' opinions seemed to be associated with the fishing techniques employed. For example, the fishers who used pole and lines were more willing to take part in the conservation of Chinese white dolphin than those who used gill nets. Among the fishers who used gill nets, only 24.2% could accept restrictions on the use of gill nets within limited ecological core zones. Increased communication should help to get more fishers using gill nets to understand the rationale behind these restriction measures. Additionally, the fishers who used pole and lines seemed to have negative impression towards gillnet fishery, since it may result in ghost fishing that caused adverse effects to the marine environment.

Coastal bottom trawling disturbed the majority of the local fishers since it caused severe damage to fishery resources and benthic ecology. Currently, the Fisheries Agency has banned bottom trawling within 3 nautical miles of Taiwan’s coastal waters to conserve nearshore habitats. However, its effectiveness has long been criticized since law enforcement capacity is inadequate and illegal fishing occurs repeatedly. After the Chinese white dolphin MWH is established in the future, the enhanced enforcement on banning bottom trawling provides a mean for coastal habitat protection and can be viewed as an incentive for fishers to support the MWH. If local fishers can comply with conservation measures and assist in law enforcement, such as reporting illegal bottom trawling when sighted, an improved conservation results should be achieved. Regarding management measures for the future Chinese white dolphin MPA, 62.9% and 50.5% of the responding fishers approved of seasonal closure and no-take reserve in selected core zones, respectively. Only 7.2% did not accept any conservation measures. Therefore, it seemed that if early consultation and communication between the government and fishing communities can be achieved, measures acceptable to fishers can be negotiated to create win-win situations.

### Future challenges

This study was conducted in 2011–2012 when the government actively delineated the habitat for the conservation of Chinese white dolphin. Although the opinions of stakeholders may progress with time, the conflict for designating Chinese white dolphin MWH still remains. Currently (as of 2016), no MPAs have been designated for marine cetaceans in Taiwan. The government's hesitation to establish Chinese white dolphin MWH seems to be attributed to its conflict with the current energy policy of installing thousands of offshore wind turbines, since MWH and turbines may compete with the same coastal waters. In contrast to China and Hong Kong, which have already designated MPAs for the Chinese white dolphin, and New Zealand and Australia, which have established MPAs for other cetacean species, Taiwan’s progress in cetacean conservation remains relatively slow [17, 33–36]. The designation of MWH for the Chinese white dolphin is the first time to establish an MPA in Taiwan. This proposed area is greater than 700 km^2^, across multiple jurisdictions of coastal cities and counties. This proposed MWH is also a milestone for Taiwan in responding to international trend of marine environmental conservation. Therefore, communication with local fishers’ associations and fishers must be further strengthened to reduce opposition from fishery sector.

The respondents from fishery sector had the relatively intense negative perception that industrial pollution was the cause of the decline in fisheries. They indicated that industrial pollution impacts both the population of Chinese white dolphin and coastal living resources. The respondents expressed that wastewater discharged in the upstream rivers was the main source of marine pollution. Researchers also indicated that the Chinese white dolphins in the waters of Hong Kong were severely influenced by the heavy metals and halogenated contaminants [37–40]. However, these external threats are hard to mitigate even if an MPA is established, since the managing authority of MPA has no jurisdiction over the pollution behavior outside its boundary. Therefore, cooperation and coordination among the MPA authority and other governmental environmental sectors is necessary to achieve an improved coastal environment. After the Chinese white dolphin MWH is legally announced in the future, environmental monitoring should be a key focus to maintain or improve coastal water quality. Not only would this facilitate the recovery of coastal living resources, but it could also provide the Chinese white dolphin a better living environment.

During the planning of the MPA, environmental education for and the participation of local residents are crucial foundational tasks [29]. The success of promoting the MPA will depend on the participation of the community [27]. Regarding conservation, communication with stakeholders in the fishery sector must continue. Ecosystem-based conservation approach must be adopted to achieve a balanced development between ecological conservation and sustainable fisheries. Dolphins are higher trophic-level consumers that their status serves as ecological indicators for the health of marine ecosystem. The idea that the conservation of the Chinese white dolphin’s habitats can simultaneously achieve the recovery of living resources must be advocated among fishers. This is a direction worth considering for the competent authority in the future communication with fishery sector.

## Supporting Information

S1 Text Questionnaire used in this study. (DOCX)
