# Supplementary material for: Conservation of the Eastern Taiwan Strait Chinese White Dolphin (Sousa chinensis): Fishers' Perspectives and Management Implications
Source: PLoS One. 2016 Aug 15;11(8):e0161321. doi: 10.1371/journal.pone.0161321 (PMC4985060; doi:10.1371/journal.pone.0161321)
Supplement: S1 Text — (DOCX) [file pone.0161321.s001.docx]

**Questionnaire**

Date： / / 2011

Dear Sir

This is a questionnaire related to the conservation of the Chinese white dolphin. Please provide your personally opinions. Your help will be deeply appreciated. Thank you for your cooperation.

Best regards,

National Cheng Kung University

Ta-Kang Liu

1.Gender：□Male　□Female

2.How many years do you engage in fishery industry？

　□below 5 years　 □5~10 years　 □10~15years　 □15~20 years

　□20~25 years □more than 25 years

3.What is your age？

　□ <20 □20~29 □30~39 □40~49 □50~59 □ >60

4.Tonnage of your fishing vessel：

□ CTS/CTX　□ CTR/CTY　□ ＜5 ton CT0　□ 5~9 ton CT1

□ 10~19 ton CT2 □ 20~49 ton CT3　 □ 50~99 ton CT4 □ >100 ton

5.What is your fishing method？

□ Gill net　 □ Trawling　 □ Long line □ Pole and line

□ Torch light net □ Beach seine □ Fixed fishing net □ Purse seine □ Other ____________

6.Have you ever sighted the Chinese white dolphin？

□ Yes

□ No【Skip to Question No 13】

7. Do you feel that there is a change in the number of Chinese white dolphins in the past 10 years when compared with 10 years before?

　□ Increase a lot □ Slightly increase □ No change □ Slightly decrease

□ Decrease a lot

8. According to your experience, what month do you see the Chinese white dolphin? (Please circle the month)

1 2 3 4 5 6 7 8 9 10 11 12 ，□ no difference

9. When you see the Chinese white dolphin nearby, does the fish landing change a lot？

□ Increase a lot □ Slightly increase □ No change □ Slightly decrease □ Decrease a lot

10. Do you think the decline of coastal fishing resources being related to the foraging of the Chinese white dolphins?

□ Closely related □ Related □ No comment □ Not related

□ Significantly not related

11. Have you ever heard of the bycatch of the Chinese white dolphin?

　 □ Always □ Usually □ Casually □ Seldom □ Never

12. Do you agree the conservation of the Chinese white dolphin?

□ Strongly agree　□ Agree　□ No comment　□ Oppose

□ Strongly oppose

13. Do you agree the government have propagated enough information on the conservation of the Chinese white dolphin?

□ Strongly agree　 □ Agree 　□ No comment 　□ Oppose

□ Strongly oppose

14. Do you agree that fishers should be involved during the planning of the Chinese white dolphin marine protection area?

□ Strongly agree　□ Agree　□ No comment　□ Oppose

□ Strongly oppose

15. During the planning stage for the Chinese white dolphin MPA, are you willing to involve in related public hearing or related meeting?

□ Yes【Jump to 18】 □ No comments　□ No

16. If you do not want to be involved, what is the reason？【Multiple choice】

□ I have no confidence in the government's policy

□ My work time will not allow me to paricipate

□ I am not interested

□ Others, please specify: __________________

17. If you have a chance, will you be willing to participate in works related to the conservation of the Chinese white dolphin?

□ Very willing　□ Willing　□ No comments　□ Not willing

□ Very Reluctant

18. Do you agree if it is appropriate to limit the use of Gill net for better conservation of the Chinese white dolphin?

□ Strongly agree　□ Agree　□ No comment　□ Oppose

□ Strongly oppose

19. Do you agree that the protection of the cetacean species is important to the marine ecology?

□ Strongly agree　□ Agree　□ No comment　□ Oppose

□ Strongly oppose

20. Do you agree that the protection of the Chinese white dolphin can facilitate the conservation of marine fishery resources?

□ Strongly agree　□ Agree　□ No comment　□ Oppose

□ Strongly oppose

21. In order to protect the marine resources, which of the following action do you want to participate?

□ Planning the marine reserves　□ Seasonal moratorium

□ Limit on the harvest □ Limit the net size within 3 nm boundary

□ None

□ Others____________

22. Do you agree that most of the fishers abide by the conservation measure prescribe by the managing authority?

□ Strongly agree　□ Agree　□ No comment　□ Oppose

□ Strongly oppose

-------------- Thank you --------------
